# Benchmarking of computational demultiplexing methods for single-nucleus RNA sequencing data

**DOI:** 10.1093/bib/bbaf371

**Published:** 2025-07-24

**Authors:** Yile Fu, Mohamad Youness, Alessia Virzì, Xinran Song, Michiel R L Tubeeckx, Gilles W De Keulenaer, Hein Heidbuchel, Vincent F M Segers, Karin R Sipido, Bernard Thienpont, H Llewelyn Roderick

**Affiliations:** Laboratory of Experimental Cardiology, Department of Cardiovascular Sciences, KU Leuven, Herestraat 49, 3000 Leuven, Belgium; Laboratory of Experimental Cardiology, Department of Cardiovascular Sciences, KU Leuven, Herestraat 49, 3000 Leuven, Belgium; Laboratory of Experimental Cardiology, Department of Cardiovascular Sciences, KU Leuven, Herestraat 49, 3000 Leuven, Belgium; Laboratory for Functional Epigenetics, Department of Human Genetics, KU Leuven, Herestraat 49, 3000 Leuven, Belgium; Laboratory of Physiopharmacology, Universiteitsplein 1, Building T (2nd Floor), 2610 Antwerp, Belgium; Laboratory of Physiopharmacology, Universiteitsplein 1, Building T (2nd Floor), 2610 Antwerp, Belgium; Department of Cardiology, ZNA Middelheim Hospital Antwerp, 2610 Antwerp, Belgium; Research Group Cardiovascular Diseases, GENCOR, University of Antwerp, 2610 Antwerp, Belgium; Department of Cardiology, University Hospital Antwerp, 2610 Antwerp, Belgium; Laboratory of Physiopharmacology, Universiteitsplein 1, Building T (2nd Floor), 2610 Antwerp, Belgium; Department of Cardiology, University Hospital Antwerp, 2610 Antwerp, Belgium; Laboratory of Experimental Cardiology, Department of Cardiovascular Sciences, KU Leuven, Herestraat 49, 3000 Leuven, Belgium; Laboratory for Functional Epigenetics, Department of Human Genetics, KU Leuven, Herestraat 49, 3000 Leuven, Belgium; Laboratory of Experimental Cardiology, Department of Cardiovascular Sciences, KU Leuven, Herestraat 49, 3000 Leuven, Belgium

**Keywords:** snRNA-Seq, donor demultiplexing, genetic variation

## Abstract

Single-nucleus RNA sequencing (snRNA-Seq) has transformed our understanding of complex tissues, providing insights into cellular composition and heterogeneity in gene expression between cells, and their alterations in development and disease. High costs however constrain the number of samples analysed. Sample pooling and their demultiplexing following sequencing based on prior labelling with antibodies or lipid anchors conjugated to DNA barcodes (cell hashing and MULTI-seq), or using genetic differences between samples, provides a solution. However, there remains no comprehensive evaluation of these demultiplexing tools to guide selection between them. Here, we benchmark the leading software (Vireo, Souporcell, Freemuxlet, scSplit) used for sample demultiplexing using genetic variants. We further compared obtaining genetic variants from SNP array analysis of gDNA and from sample-matched bulk-RNA-Seq data, identified using three different variant calling tools (BCFtools, cellSNP, FreeBayes). Demultiplexing performance was evaluated on simulated multiplexed datasets comprising two, four, and six samples with doublet percentages between 0% and 30%, and validated against demultiplexing using sex-linked genes. Software implementation and execution were evaluated by run speed, robustness, scalability, and usability. Our results show that all tools excluding scSplit provide high recall and precision with an accuracy of 80%–85%. Vireo achieved the best accuracy. Demultiplexing tools were differentially affected by the variant calling tool with which it was paired. For all tools, accuracy decreased with the increasing percentage of doublets. Deployment of demultiplexing during analysis of pooled real-world 10x RNA-Seq data from the human heart and from different species is shown, as are advantages for doublet detection and removal.

## Introduction

Droplet-based single-nucleus sequencing (snRNA-Seq) has revolutionized biomedical sciences by revealing cell-specific, transcriptome-wide gene expression levels within complex tissues [1–4]. This technology has provided new insights into cell heterogeneity, cell differentiation trajectories, and cell–cell communication mechanisms. However, the high costs of performing such analysis can lead to compromises in experimental design including limiting the number of biological replicates studied, reducing power to detect effects. Such compromises are particularly problematic in the analysis of samples with high biological variability such as in patient samples and in the detection of small effects. Data dropout is also exacerbated by low sample numbers, increasing the likelihood of false discoveries. Increasing biological replicates through sample pooling prior to library preparation followed by demultiplexing (i.e., identifying and isolating the sequencing data related to each sample within the pool) to the original biological sample is an effective strategy to overcome these issues as well as reduce batch effects [5–9].

The feasibility of pooling samples prior to droplet-based sequencing to later demultiplex was illustrated in so-called barnyard experiments, whereby sequencing libraries generated from samples of mixed species were demultiplexed using species genomes [10–15]. To solve the greater challenge of demultiplexing samples coming from the same species, two main approaches have been deployed: 1, Cell hashing, which involves labeling of cells or nuclei from each sample with genetic barcodes via antibodies, lipids anchors, or ectopic expression; 2, Bioinformatic demultiplexing using the natural genetic variation between samples as unique identifiers. Each of these approaches comes with its own advantages and disadvantages. While cell hashing has the advantage that no a priori knowledge of the sample is required, the labeling of cells/nuclei with sample-specific tags such as for MULTI-Seq, requires significant optimization to ensure specific labeling. Additional sample processing steps may also decrease throughput and compromise sample quality, while the additional reagents required increase costs [16–18]. More importantly, although when applied to live cells these methods can achieve >90% demultiplexing accuracy (defined as the number of all matching singlets plus the number of matching non-singlets divided by the total number of cells), accuracy is lower on nuclei isolated from archived/frozen samples (snRNA-Seq) [15, 19, 20]. For bioinformatic demultiplexing using the natural genetic variation between individuals, some issues associated with cell hashing such as inefficient labelling and extended processing times are overcome. However, genetic information (such as single-nucleotide polymorphisms [SNPs]) is required. This can be obtained from genetic variants expressed in the RNA-Seq dataset to be demultiplexed. For example, Vireo [7], Souporcell [21], Freemuxlet [22], and scSplit [8] infer the genotype directly from the snRNA-Seq reads. However, in the absence of the genetic variant profile of each sample, these demultiplexing tools cannot assign the demultiplexed individual transcriptomes in the pool analysed to the originating sample. Rodent strain-specific SNPs [23] or those that are present in the human population (e.g. 1000 Genomes) available in publicly accessible databases can also be used to distinguish samples from pooled libraries, but as for expressed variants, do not allow assignment to specific individuals. To assign demultiplexed samples to their original sample, sample-specific information must be obtained via genomic DNA analysis, e.g. SNP arrays (providing a reference genotype), or from parallel RNA-sequencing data of each sample. Indeed, taking this approach, Demuxlet, one of the first bioinformatic tools developed, showed that as few as 50 genetic variants were determined to be required to separate up to 64 samples [9]. Despite these additional steps to obtain genetic information, the potential for high accuracy and the option to sequence nuclei make demultiplexing using SNPs a preferred option when archived human tissue samples are to be analysed. However, while each of the approaches and their associated tools outlined above perform well under certain metrics, a systematic third-party benchmarking of available and widely used tools for their demultiplexing accuracy and computational efficiency is lacking. Moreover, how these tools perform in demultiplexing snRNA-Seq, which is the methodology of choice for single-cell sequencing of complex solid tissues, such as the heart, is not determined. In the absence of such data, informed decisions based on real-world findings that can be deployed to optimize experimental outcomes are not possible.

The ability to identify different sample source of cell transcriptomes has also been applied to solve the issue of cell doublets that occur in droplet-based approaches, whereby 2 nuclei are encapsulated in a single GEM (droplet) generating a single library. These doublets may comprise 2 nuclei of the same cell type (homotypic) or may be of different cell types (heterotypic) and may together constitute as many as 40% of droplets [24]. The prevalence of doublets is particularly increased in protocols involving increased cell/nuclear analysis (overloading) during sequencing [25]. Without identification and removal of doublets, cell type clustering, differential gene expression analysis, and inference of cell developmental trajectories are severely compromised [26, 27]. While a number of tools have been developed to remove doublets in 10x RNA-Seq pipelines, these function to remove heterotypic doublets by comparing the transcriptional profile of each droplet to a simulated doublet model derived from the dataset. Through introducing additional genetically distinct nuclei into the sequencing pipeline by sample pooling prior to GEM formation, the proportion of doublets that are heterogenic in composition is increased proportionately with increased number of samples pooled (from genetically different samples). These heterogenic doublets may then be specifically identified using demultiplexing tools, increasing the accuracy of doublet exclusion from downstream analysis combined with doublet detection tools, such as DoubletFinder [28].

To provide a reliable pipeline capable of accurately demultiplexing pooled snRNA-Seq data, we performed a benchmark evaluation of genetic variation-based demultiplexing in snRNA-Seq data generated on human cardiac tissue. To this end, we used *in silico* simulations constructed from 10x experimental snRNA-Seq datasets with known sample identity for each nucleus. We evaluated four widely used demultiplexing methods: Vireo, Souporcell, Freemuxlet, and scSplit (we excluded Demuxlet because Freemuxlet is the SNP-free version of Demuxlet) and two strategies for selecting the genotype reference list of SNPs, SNP array-derived and the matched bulk RNA-Seq-based SNPs. We also included three of the best-performing variant calling tools (BCFtools [29, 30], cellSNP [31], and FreeBayes [32]) to generate different pipelines [33–35]. We evaluated these methods/pipelines based on their overall demultiplexing accuracy in real-world and simulated data with varying doublet proportions. We also compared the computational efficiency of the demultiplexing methods in terms of their speed, scalability, robustness, and usability. The pipeline of demultiplexing based on species differences was also examined.

Relative to the best-case scenario with species or sex differences, we found that apart from scSplit, each demultiplexing method performed well across all evaluation criteria. Irrespective of the source of genetic information, demultiplexing accuracy was not influenced by the number of samples pooled but was affected by doublet proportion. Vireo and scSplit consistently achieved the best and poorest performance, respectively. Vireo also performed the best in terms of speed and scalability. Based on the proportion of reads of each origin, the species-mixed dataset was successfully demultiplexed when mapping to the genome. Overall, Vireo is highlighted as the best computational demultiplexing method for its highest demultiplexing accuracy and for being computationally efficient. To aid other investigators in their analysis, implementation of Vireo in a standard pipeline for snRNA-Seq analysis is shown.

## Methods

### An expanded methods section is available in the supplementary materials online

#### Human samples for snRNA-sequencing data

Left ventricular biopsies were collected in our study of human heart failure under the protocol approved by the ethical committee of UZ Leuven (S58824), and implemented within the UZ Leuven heart transplant program. Atrial tissue was collected during surgery at the Department of Cardiology, University Hospital Antwerp, Antwerp, Belgium. Informed consent was obtained from all patients. The study was approved by the institutional review committee of the University Hospital Antwerp, and the procedures followed were in accordance with institutional guidelines (EDGE001634). Both study protocols conformed to the Helsinki Declaration and were conducted according to national and European Union regulations on the use of human tissues. Patient information is provided in Supplementary Table S1. Tissue samples were taken and snap-frozen in liquid nitrogen and stored at − 80°C.

### Benchmark evaluations workflow

The performance of demultiplexing methods was tested on 10x snRNA-seq datasets generated in house, which were incorporated into simulated multiplexed datasets (2 samples, four samples, and six samples) using the Snakemake [36] workflow with modifications [37]. Simulated datasets were generated with doublet rates of 0%, 10%, 20%, and 30%. 10x snRNA-Seq was performed on nuclei prepared from frozen human ventricle or atrial tissue. Sequencing reads of individual samples were aligned to the GRCh38 reference genome and processed with 10x Genomics Cell Ranger 7.0.0 [38]. Candidate demultiplexing methods/pipelines were applied to the simulated dataset using either SNP array-derived or matched bulk RNA-Seq-derived genetic variation. A schematic of the benchmarking pipeline is provided in Fig. S1 available online at http://bib.oxfordjournals.org/, and a summary of the demultiplexing methods and different datasets used in this study is in Table 1 and Supplementary Table S2, respectively.

**Table 1:** Overview of the four demultiplexing software tools evaluated in this study.

Method	Programming language	Version	The time of release	Software description
Vireo	Python	0.2.3	2019	Demultiplexing pooled scRNA-Seq data with or without genotype reference
Souporcell	Python, Rust	2.0	2020	Clustering scRNA-Seq data by genotypes
Freemuxlet	C++, C	0.1	2019	Software in the suite of population-scale analysis tools for single-cell genomics data
scSplit	Python	1.0.9	2019	Genotype-free demultiplexing of pooled scRNA-Seq

### Benchmark environment and parameter settings

All demultiplexing methods were executed on a server with two Intel(R) Xeon(R) Platinum 8360Y CPUs, 1024 GB of memory, and a CentOS 7.9 system. The parameters of demultiplexing methods were set to their recommended values or default values if no recommendation was available. The latest version of each method (August 2023, Table 1) was used.

### Scalability, robustness and usability

Each demultiplexing method/pipeline was applied to each combination of 2, 4, and six sample datasets with 0%, 10%, 20%, and 30% doublets. The relationship between pipeline run time and the number of samples was plotted to show the scalability, and the distributions of the mean precision and recall across subsets were plotted to show robustness, respectively.

Each demultiplexing method was evaluated as excellent, good, and fair (1, 0.5, and 0, respectively) based on four criteria: software quality, execution convenience, publication, and documentation & support. Software quality indicates whether a demultiplexing method can be executed on all simulated and real-world datasets used in this study. ‘Execution convenience’ relates to the popularity of the computational approach required to run a method. Methods written in R and Python packages are preferred owing to their wider use in the community. ‘Publication’ relates to whether a demultiplexing method has been published in a peer-reviewed journal. ‘Documentation & Support’ evaluated a method’s user-support resources, such as open-source code, tutorials, and active Q&A.

## Results

### Use of sample-specific genetic variation obtained from SNP array for demultiplexing of pooled snRNA-Seq data

We first tested the performance of four tools to demultiplex individual samples from pooled snRNA-Seq datasets using sample-specific SNPs (total SNPs, including genic SNPs), which were obtained from gDNA using the Illumina Infinium Omni2.5–8 bead array (Table 2). Since samples were mixed for sex, sex chromosome-based SNPs were excluded.

**Table 2:** Number of genetic variants detected by different methods in each sample.

Sample	SNP Array (Total/Genic)	Transcriptome		
		BCFtools (Total / Genic)	FreeBayes (Total / Genic)	cellSNP (Total / Genic)
Donor 1	2,567,717/121,742	87,707 / 24,289	222,736 / 87,738	1,557,299 / 1,372,536
Donor 2	2,567,717/121,742	82,067 / 26,691	158,917 / 77,104	1,357,357 / 1,259,277
Donor 3	2,567,717/121,742	77,196 / 24,347	191,861 / 90,175	1,410,792 / 1,286,571
Donor 4	2,567,717/121,742	76,299 / 25,515	144,512 / 71,332	1,240,785 / 1,119,940
Donor 5	2,567,717/121,742	77,899 / 25,872	127,870 / 71,430	1,177,396 / 1,107,295
Donor 6	2,567,717/121,742	77,994 / 26,897	130,003 / 68,778	1,222,546 / 1,142,823

^*^Values are presented as total SNPs/SNPs within transcribed genes.

**Figure 1:**
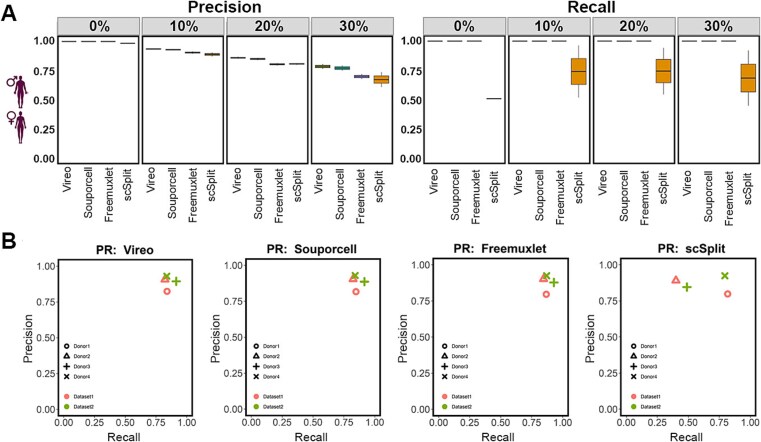
Evaluation of four demultiplexing methods using SNPs derived from arrays on two sample datasets. The precision (left) and recall (right) obtained using each demultiplexing software when used on two sample datasets (A) under 0%, 10%, 20%, and 30% doublet rate, which is the percentage of doublets in each simulated dataset. (B) Performance evaluations of four demultiplexing tools on two real-world datasets. Each dataset contains two samples, one male and one female, respectively. The ground truth is referred to as the prediction based on the sex chromosome. Performance is evaluated in terms of precision (y-axis) and recall (x-axis).

To test pipeline performance as well as its validation, demultiplexing tool accuracy (Precision - the proportion of identified nuclei for each sample that are true singlet nuclei from the correct donor, and Recall - the proportion of true singlet nuclei for each sample that are identified as singlets and assigned to the correct donor) was first assessed on simulated pools of 10x snRNA-Seq datasets, which we had generated for individual human cardiac ventricular samples. The capacity of the pipelines to demultiplex 2 pooled samples was first evaluated (Table 3). Since in practice, snRNA-Seq datasets include doublets at varying proportions, we further evaluated how the ability of these tools to reliably demultiplex the nuclei to the original donor was affected by different doublet percentages, which we simulated at 0%, 10%, 20%, and 30% for each pool. The doublets and singlets for each scenario are summarized in Table 4 and Supplementary Table S3. The potential types of doublets are illustrated in Fig. S2 available online at http://bib.oxfordjournals.org/. Generated doublets comprised each combination of samples. However, only the heterogenic doublets, comprising half of all doublets, would be detectable by the pipeline. At 0% doublets, the demultiplexing tools exhibited precision ranging from 100% to 98%, which gradually decreased with increasing doublets to the lowest precision of 70% obtained with Scsplit. Recall that all doublet percentages was 100% for all tools other than ScSplit, which had a median of 52% (Fig. 1A).

**Table 3:** Number of snRNA-Seq samples and number of nuclei per sample for each dataset.

Dataset	No. of sample	Sample ID	No. of nuclei
Dataset 1	2	Donor 1	6281
		Donor 2	5866
Dataset 2	4	Donor 3	4483
		Donor 4	5929
		Donor 5	6743
		Donor 6	7489
Dataset 3	6	Donor 1	6281
		Donor 2	5866
		Donor 3	4483
		Donor 4	5929
		Donor 5	6743
		Donor 6	7489

**Table 4:** Number of snRNA-Seq doublets for each scenario in each dataset.

Dataset	Scenario	Singlets	Doublets
Dataset 1	0%	12,147	0
	10%	11,043	1105
	20%	10,122	2026
	30%	9344	2804
Dataset 2	0%	24,644	0
	10%	22,404	2241
	20%	20,537	4108
	30%	18,957	5688
Dataset 3	0%	36,791	0
	10%	33,446	3346
	20%	30,659	6133
	30%	28,301	8491

Having shown successful demultiplexing of a simulated pool of two individual samples, we tested the efficacy of this approach for demultiplexing RNA-Seq data that was generated on two human ventricular samples that were pooled prior to library generation. The use of this real-world data would allow us to test the performance of the demultiplexing tools in the presence of bona fide doublets and ambient RNAs. To these ends, 10x RNA-Sequencing was performed on ventricular samples from one male and one female, which were pooled after tissue homogenization before library generation. The demultiplexing performance of the four tools was then tested based on sex chromosome-associated genes, which were used as a ground truth. 579 Y chromosome genes were extracted from the Ensembl database and used. Each nucleus was then assigned to the male or female sample according to the expression or absence of each Y chromosome gene.

As shown in Fig. 1B, using the array-derived SNP for demultiplexing, Vireo, Souporcell, and Freemxulet successfully disaggregated each sample within this real-world dataset. Precision and recall values of between 85% and 90% were obtained with these three tools, with only minor differences between them detected. In contrast, scSplit performed poorly across the two datasets (Fig. 1B). The recall of scSplit was less than 50% on the assignment among the two samples in each dataset while the precision was circa 80%.

We next extended our analysis to more complex pools of four and six samples. Given that 12-16 k nuclei can be analysed per lane of a 10x Chromium microfluidics chip, this number of samples would balance sample throughput with cost and nuclei analysed per sample. Simulated pooled datasets comprising four and six of the individual datasets (Table 3) were created and SNPs were obtained for the respective samples (Table 2).

**Figure 2:**
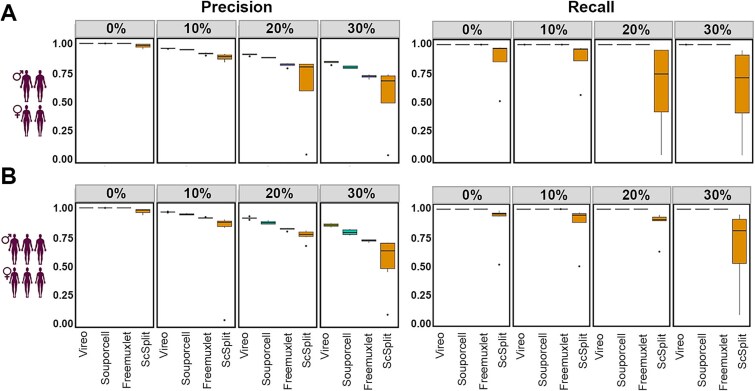
Evaluation of demultiplexing methods using SNPs derived from array to detect genetic variance on pooled datasets from four and six patients. (A) the precision (left) and recall (right) achieved when analysing datasets of four pooled samples with 0%, 10%, 20%, and 30% doublets. (B) As in a with six samples.

Demultiplexing was tested for each of the four doublet percentages. At 0% doublets, as observed for two samples, all tools successfully demultiplexed the four and six samples, achieving close to 100% precision. With an increasing percentage of doublets, demultiplexing precision decreased for all tools, with scSplit showing the greatest decline in performance, achieving a median precision of ~70% at 30% doublets (Fig. 2A, B). We observed only subtle differences between median precision values for each method when applied to four and six samples, suggesting the performance of the demultiplexing method is more sensitive to doublet number rather than the number of samples multiplexed (Fig. 2A, B). Recall also exhibited a similar result in the four and six sample datasets compared to the two sample datasets (Fig. 2A, B). The recall value obtained by all methods except for scSplit was close to 100%, even with 30% doublets.

Together, these analyses of demultiplexing precision and recall over 2–6 samples and on real-world data indicate a superior performance for Vireo and Souporcell on sample demultiplexing using genetic information obtained from SNP arrays.

Use of sample-specific genetic variation obtained from bulk RNA-Seq for demultiplexing of pooled snRNA-Seq data.

As a second approach to identify patient-specific sequence variants for demultiplexing, genetic variants expressed in the transcriptome were used for demultiplexing. The relatively low cost of bulk RNA-Seq, similar to SNP array, makes this a viable method of detection of individual genetic variation. Bulk RNA-Seq at a depth of ~50 million reads per sample was carried out on poly-A isolated mRNA from tissue samples, matched with those used for 10x RNA-Seq. The variant calling software tools BCFtools, cellSNP, and FreeBayes were then used to identify expressed sequence variants. The number of genetic variants detected by each software tool is summarized in Table 2. These tools were chosen since probabilistic method-based tools performed best in previous benchmarking studies [33, 37]. Given the lower number of SNPs detected, scSplit was excluded from this comparison owing to the low accuracy we observed in the demultiplexing analysis using SNPs.

**Figure 3:**
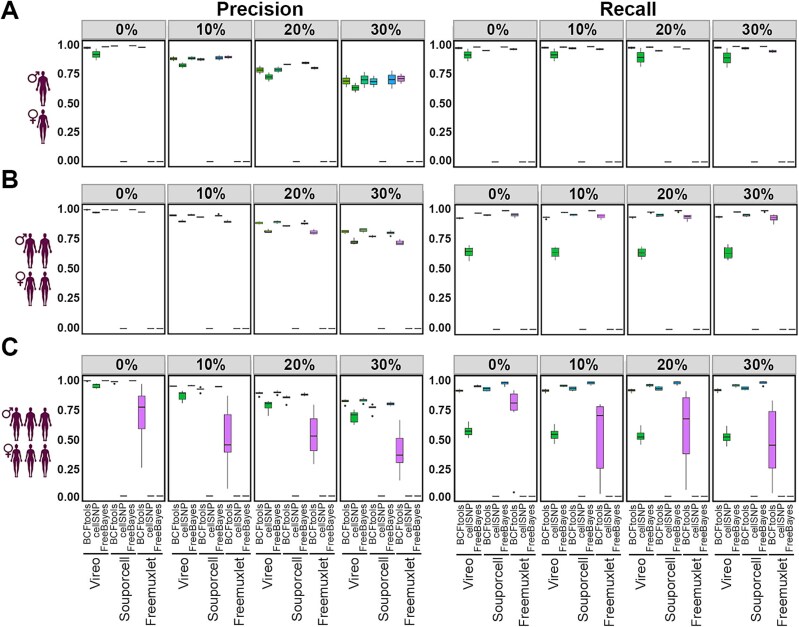
Evaluation of demultiplexing pipelines using SNPs/expressed variants derived from sample matched bulk RNA-Seq data as source of genetic variation on simulated datasets. (A) The precision (left) and recall (right) achieved using each permutation of demultiplexing and variant calling tool for demultiplexing two sample datasets with 0%, 10%, 20%, and 30% doublets. The precision and recall were set to zero to reflect the pipeline’s inability to produce meaningful predictions. (B) As in a for four samples. (C) As in a for six samples.

The performance of each permutation of the three demultiplexing and three variant calling tools (9 pipelines in total) to disaggregate pools of 2, 4, and six samples with doublets ranging from 0 to 30% was tested. The results of these trials showed clear differences in performance, ranging from high precision, approaching 100%, to as low as 0% precision (pipeline failures were set to zero). As a general pattern, the best-performing pipelines for two samples were also best at demultiplexing 4 and 6 samples. A decrease in precision was also typically detected as doublet percentage increased but recall was less affected (Fig. 3A). Specifically, while recall performance on four datasets was lower than for the two sample datasets, it remained above 95%, except for the combination of Vireo with cellSNP, which had a recall of 60% (Fig. 3B). Moreover, the precision of all pipelines decreased to 75% when doublets were set to 30% (Fig. 3C). This relationship was consistent with that observed in the demultiplexing trials in which SNPs derived from arrays were used (Fig. 2A, B).

All pipelines that included cellSNP had an inferior performance to those which included the other variant calling tools. While at 0% doublets, cellSNP combined with Vireo performed with a precision and recall of 90% and 88% respectively (Fig. 3A), when used in combination with Souporcell and Freemuxlet, precision and recall were both 0. For the two sample pool, Freemuxlet in combination with FreeBayes also had a precision and recall of 0 (Fig. 3A). As the sample number increased from two to four and 6, the performance of cellSNP in combination with Vireo decreased further, particularly in recall (Fig. 3B, C). Moreover, although the BCFtools-Freemuxlet combination exhibited a comparable performance to other pipelines for two and four samples, the performance of this pipeline dramatically decreased for six samples in terms of precision and recall across all doublet percentages (Fig. 3C). A possible reason for this discrepancy is the number of variants and the format of the SNP. The number of variants called by BCFtools was insufficient to discriminate the difference between samples in the six samples dataset. Therefore, some samples in the dataset were assigned to the wrong donor. Indeed, the strong correlation between the number of variants detected by the calling tools with demultiplexing performance supports the importance of variant number in the capacity of the demultiplexing tool to perform its function.

### Effect of number of samples in the sequenced pool on demultiplexing performance

In Figs. 1–3, we demonstrated the performance of the various demultiplexing pipelines for demultiplexing of 2, 4, or 6 samples. However, while this analysis showed how each pipeline performed against its competitors, it did not illustrate how sample pool complexity affected each pipeline individually. To obtain this information, we re-plotted the data from Figs. 1–3, whereby the sample number was plotted against accuracy individually for each method/pipeline.

**Figure 4:**
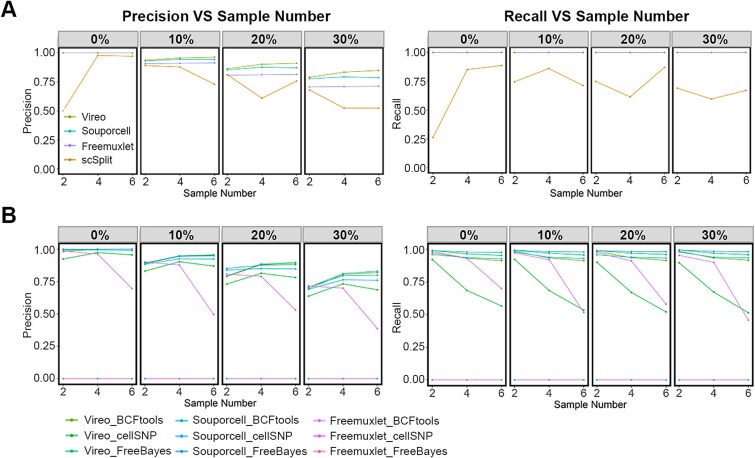
Comparison of performance of demultiplexing methods/pipelines across pools of 2–6 samples. Distributed performance (precision, left; recall, right) of each method across two, four, and six samples under different doublet rates (0%, 10%, 20%, and 30%) using SNPs derived SNP array (A) and from matched bulk RNA-Seq (B). The precision and recall were set to zero to reflect the pipeline’s inability to produce meaningful predictions.

For demultiplexing using genetic variation obtained from the SNP array, with the exception of scSplit, increasing sample number did not significantly affect the accuracy of each pipeline. Moreover, increasing sample number did not exacerbate the negative influence of doublets on precision. Notably, the precision obtained using Vireo increased as the sample number increased from two to six (Fig. 4A). For demultiplexing using variants extracted from RNA-Seq, we observed a similar accuracy. For the best-performing pipelines (i.e., Vireo combined with BCFtools or FreeBayes), demultiplexing accuracy was increased with sample number under the same doublet rate, with an even greater improvement relative to other tools when a higher doublet percentage was analysed (Fig. 4B). The Freemuxlet BCFtools pipeline was an exception to this finding, showing a poorer performance as the sample number increased. Notably, the performance of Vireo combined with cellSNP showed a superior precision to other tools, which improved further as the sample number increased, while the recall value of this pipeline decreased with the sample number (Fig. 4B). Although pipelines that included SNPs identified by array and in bulk RNA-Seq generally showed similar precision, apart from for ScSplit, recall was lower when SNPs were obtained from bulk RNA-Seq. A potential reason for this difference was the number of SNPs identified. However, while the number of SNPs from RNA-Seq was not as high as from the array, they exceeded the minimum number reported necessary for demultiplexing [9]. Moreover, scSplit, which had the worst performance when included in the demultiplexing pipeline, generated the greatest number of SNPs from RNA-Seq data of the variant calling tools (Fig. S3 available online at http://bib.oxfordjournals.org/).

### Computational efficiency, robustness, scalability, and software implementation of demultiplexing methods

Since access to computing infrastructure and expertise differs between users, we also assessed the performance of the tools in terms of their computational efficiency, robustness, scalability, and software implementation.

**Figure 5:**
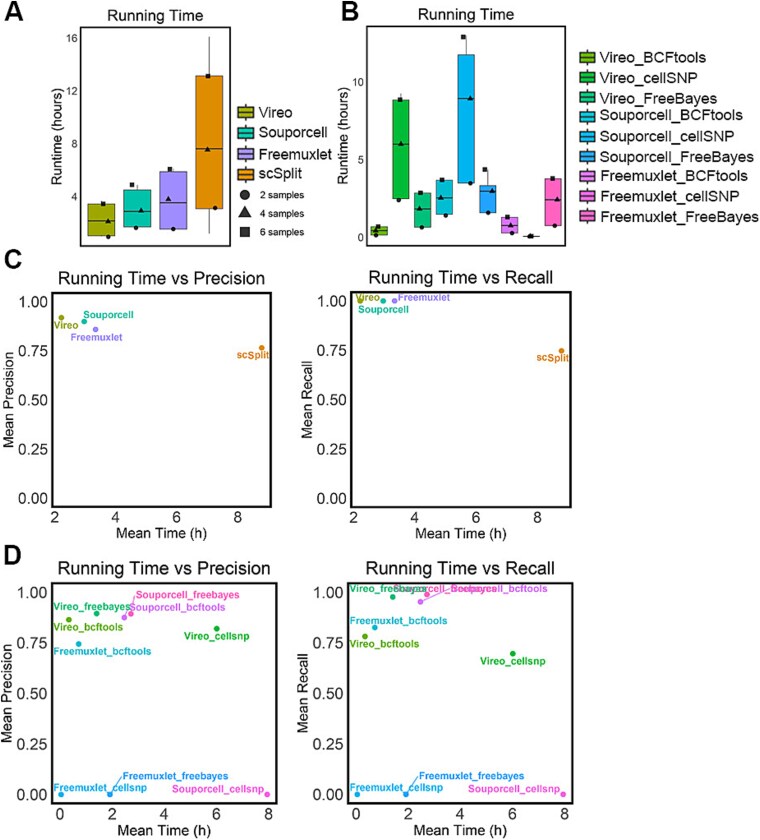
Comparison of demultiplexing methods/pipelines regarding run speed. (A) Run time of each method based on SNP array-based genetic variance. (B) Run time of each pipeline based on matched bulk RNA-Seq-based genetic variance. The run time of each tool/pipeline across two, four, six samples with 20% doublets was shown in A and B. The symbol shape is applied to A and B. (C) Mean precision (left) and mean recall (right) versus mean run time achieved across 12 datasets for each demultiplexing method based on SNPs derived from arrays. (D) Mean precision (left) and mean recall (right) versus mean run time across 12 datasets of nine demultiplexing pipelines based on matched bulk RNA-Seq-based genetic variance.

First, we compared the run time of the four demultiplexing methods. These analyses were performed for their use when using SNPs from SNP array and also using SNPs obtained from bulk RNA-Seq, where the time of the variant calling step was included in the run time (Supplementary Table S4 available online at http://bib.oxfordjournals.org/). The box and whisker plots represent the run time of all analysis scenarios (2, 4, six samples with 0%, 10%, 20%, 30% doublets) and symbols represent example analysis for 2, 4, and six samples with 20% doublets (Fig. 5A, B). For the SNP array-based method, Vireo, Souporcell, and Freemuxlet all had a similar run time of around 2 hours per pooled library while scSplit took a significantly longer time of ~8 hours (Fig. 5A). For demultiplexing using SNPs from bulk RNA-Seq, BCFtools combined with Vireo or Freemuxlet were two of the fastest pipelines, taking less than 2 hours to generate output (Fig. 5B). cellSNP combined with Vireo or Souporcell took the longest time to demultiplex. Regarding the relationship between accuracy and mean run time of the demultiplexing pipeline (the mean calculated across two to six sample datasets), while Vireo achieved the greatest accuracy (precision and recall) and was the least computationally intensive method. scSplit was not only the most computationally intensive method but also exhibited the lowest precision and recall of all the methods (Fig. 5C). However, for the bulk RNA-Seq-based pipeline, all methods performed similarly. Among all pipelines, Vireo combined with FreeBayes achieved the highest accuracy in precision and recall (Fig. 5D), while not being the most computationally efficient method. Of note, Vireo combined BCFtools was the computational efficiency method in bulk RNA-Seq-based pipeline.

**Figure 6:**
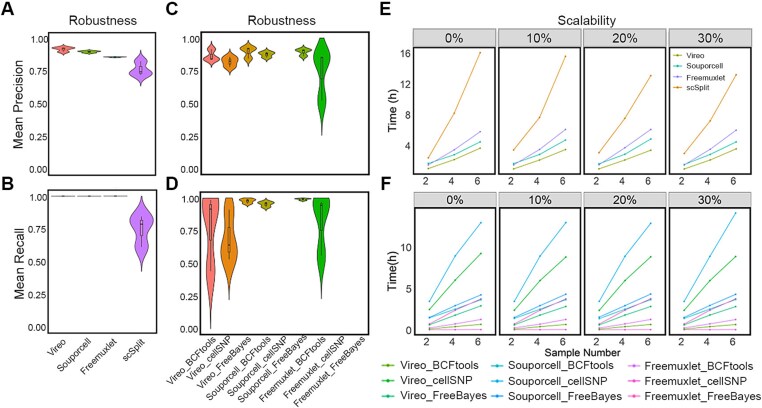
Comparison of robustness and scalability of each demultiplexing method. (A-B) Robustness of mean precision and recall of each method based on array-based genetic variance. (C-D) Robustness of mean precision and recall of each pipeline using RNA-Seq-based genetic variance. (E-F) Scalability of each pipeline measured for pipelines incorporating array-based and RNA-Seq-based genetic variant calling, respectively. Scores for the failed pipelines were set to zero.

We next evaluated the robustness of the demultiplexing methods, i.e., the sensitivity of the precision and recall achieved using the tool to variation in sample number and doublet percentage (Fig. 6). The smaller the variation, the greater the robustness. We applied each demultiplexing method/pipeline to our datasets comprising two to six samples and recorded the mean precision and recall values (scSplit was excluded from the bulk RNA-Seq-based evaluation, because of low accuracy). We generally observed that the tools (i.e., Vireo) with the highest demultiplexing precision showed the least sensitivity to sample number and doublet percentage (Fig. 6A, B). However, Freemuxlet had a significantly lower variation in precision than Vireo but it had a much lower overall accuracy. For the pipelines relying on SNPs extracted from bulk-RNA-Seq, due to some failed scenarios, which unsuccessfully assigned the cells to their original samples, the variation in their mean precision and recall was high (Fig. 6C, D). The most robust of these pipelines was Vireo combined with FreeBayes, Souporcell combined with BCFtools, and Souporcell combined with FreeBayes.

Next, we examined how run time was affected by sample number, and thus whether the pipelines were scalable. scSplit was excluded from bulk RNA-Seq-based derived SNPs analysis, because of its poor performance. All methods that used genetic variants from the SNP array had run times that scaled linearly with the number of samples (Fig. 6E). Notably, the run time of scSplit increased more rapidly with sample number than for that of the other methods. Pipelines involving SNPs obtained from bulk RNA-Seq also scaled linearly in their run time, and run time was not influenced by doublet percentage (Fig. 6F).

**Figure 7:**
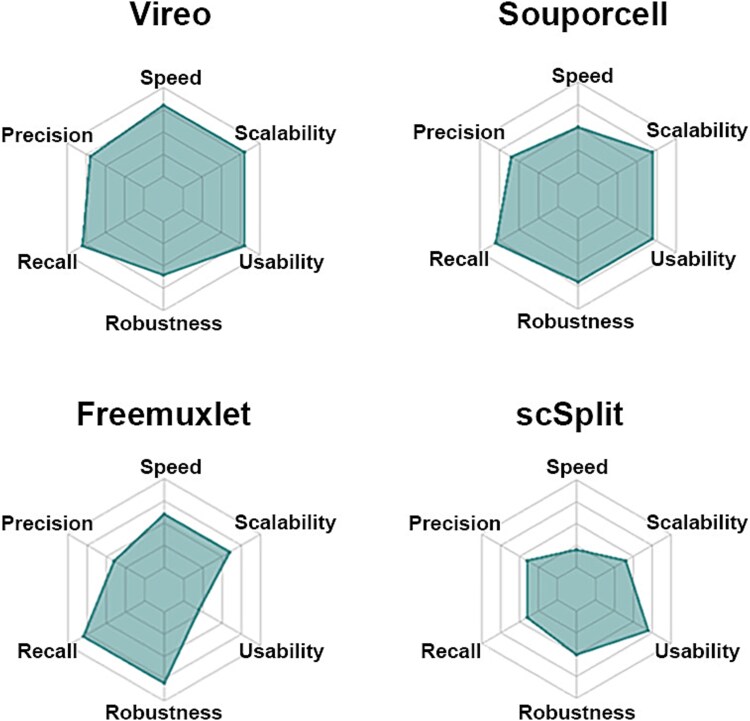
Graphical summary of benchmark results. Two aspects (precision and recall) are related to demultiplexing accuracy and four aspects (speed, robustness, scalability, and usability) are related to software implementation.

Finally, we evaluated the ease of implementation of the demultiplexing software tools. This was evaluated based on user-friendliness, software quality, and active maintenance, which are crucial to the success of bioinformatics tools. We scored each method according to its software quality, execution convenience, publication, and documentation and support. Table 5 lists our score reasoning and the overall usability score of each method. Regarding user support, all four methods have active Q&As on their software web pages for collecting users’ feedback and answering users’ questions. Among the four methods, Vireo achieved the highest usability score owing to its excellent implementation. This analysis is summarized in Fig. 7 and shows Vireo and Souporcell to be the top two methods. While these tools were scored similarly for most criteria, Vireo had a higher run speed than Souporcell. Freemuxlet had less optimal usability and precision, but its other properties (e.g. recall and robustness) were good. scSplit was the worst performing tool being inferior across all measures than the other methods.

**Table 5:** Usability of the four demultiplexing methods.

	Software Quality	Execution Convenience	Publication	Documentation & Support	Usability Score
Vireo	Excellent (success on all combinations)	Excellent (Python module)	Excellent (published in a peer-reviewed journal)	Excellent (documentation, GitHub webpage, activate Q&A)	4
Souporcell	Good (failure on one combination)	Excellent (App container)	Excellent (published in a peer-reviewed journal)	Excellent (GitHub webpage, activate Q&A)	3.5
Freemuxlet	Fair (failure on two combinations)	Fair (software in a suite)	Fair (GitHub webpage)	Excellent (GitHub webpage, activate Q&A)	1
scSplit	Good	Excellent (Python module)	Excellent (published in a peer-reviewed journal)	Excellent (GitHub webpage, activate Q&A)	3.5

We measured the usability of each method in four aspects: software quality, execution convenience, publications, and documentation and support. Each aspect has three levels: excellent, good, and fair, which correspond to scores 1, 0.5, and 0, respectively. The usability score of methods is the sum of its four scores under four aspects.

### Implementation of a demultiplexing pipeline using SNPs obtained from array analysis of individual samples into a pipeline for analysis of 10x snRNA-Seq data

**Figure 8:**
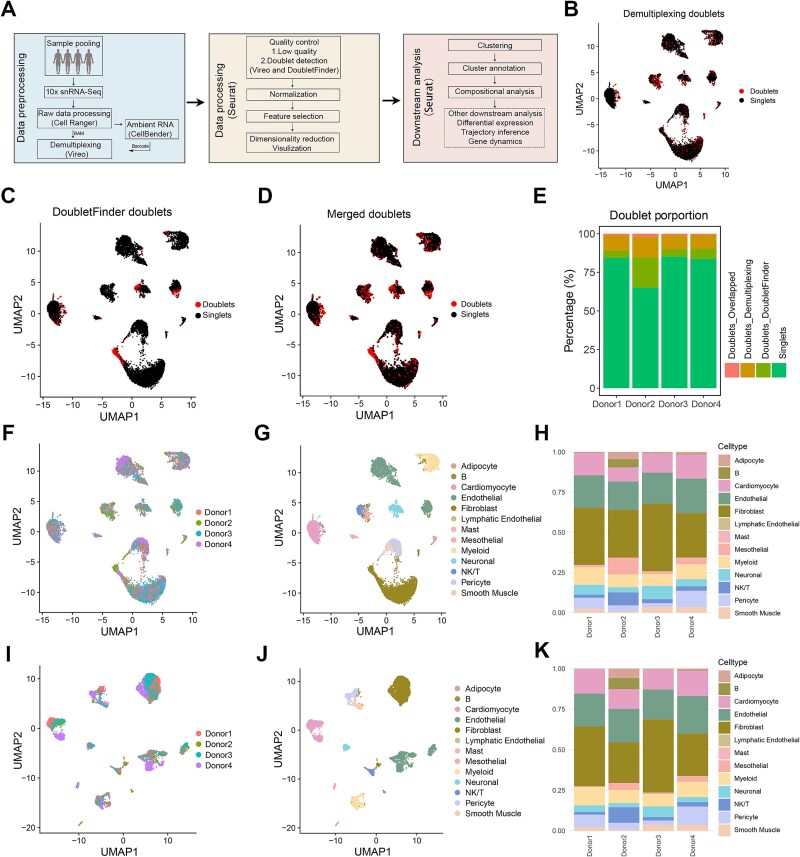
The application of Vireo to 4-sample multiplexed 10x RNA-Seq data. (A) Schematic showing the study workflow. (B) Uniform manifold approximation and projection (UMAP) representation of 1097 heterogenic doublets identified by Vireo. Doublets are highlighted in red. (C) UMAP representation of 813 heterotypic doublets identified by DoubletFinder. Doublets are highlighted in red. (D) UMAP representation of 1804 heterotypic and heterogenic doublets. Doublets are highlighted in red. (E) The stacked bar plot depicting the proportion of doublet type in each donor in the pool. (F) UMAP shows the cell distribution of each donor before removing the doublets. (G) UMAP representation of 13 cell types annotated by Azimuth before removing doublets (nuclei number: 10,836). (H) The stacked bar plot depicting the cell-type composition of each donor before removing doublets. The color coding reflecting cell types in (G). (I) UMAP showing the cell distribution of each donor after removing the doublets. (J) UMAP representation of 13 cell types annotated by Azimuth after removing the doublets (nuclei number: 9032). (K) The stacked bar plot depicts the cell-type composition of each donor after removing the doublets. The color coding reflecting cell types in (J).

Our analysis established Vireo as the best overall method for demultiplexing (Fig. 7). To illustrate how this tool can be utilized experimentally, it was integrated into a 10x snRNA-Seq pipeline where it was used to deconvolve a multiplexed dataset of four human heart samples as well as to identify and exclude doublets (Fig. 8A). The 10x RNA-Seq dataset was generated on nuclei isolated from equal masses of four cardiac tissue samples pooled prior to homogenization, flow sorting and 10x library generation on 1 lane of a 10x Chromium 3′ microfluidic chip. After the generation of the count matrix using Cell Ranger, CellBender was used to remove ambient RNA. We then deployed Vireo to perform demultiplexing using the BAM and barcode file and SNPs obtained from each sample in the pool from the Illumina Infinium Omni2.5–8 bead array. Next, we used the Seurat package to perform the data processing. The demultiplexing information from Vireo was added for each nucleus. Low-quality nuclei, identified as having <200 genes expressed, and > 5% of reads of mitochondrial genome origin were removed. In addition to the standard removal of heterotypic doublets using DoubletFinder, heterogenic doublets were also identified and removed by Vireo (Fig. 8B, C). Illustrating the added advantage of sample pooling and demultiplexing in doublet identification, 1097 additional doublets were identified by Vireo (10.12%) to 813 heterotypic doublets identified by DoubletFinder (Fig. 8D). The doublet proportion in each sample in the pool can be as high as 32.5% (Fig. 8E). When the doublets were retained in the dataset, the different cell types annotated by using Azimuth mapping onto a reference were clustered together (Fig. 8F, G). We noticed the cardiomyocyte population also included cells that were labeled as neuronal cells and fibroblasts (Fig. 8G). After doublet removal, the data were re-clustered and clusters were annotated according to cell types. Nuclei from each sample were present in each of the clusters (Fig. 8I). This not only indicates the similarity of the four tissue samples analysed but also highlights the advantage of sample pooling prior to nuclear isolation, which minimizes the risk of batch effects. Projection of the data after doublet removal onto a reference again, using the Azimuth package, identified 13 cell types, which mainly comprised cardiomyocytes, fibroblasts, and endothelial cells (Fig. 8J, 8K). After the removal of misannotated cell types, non-cardiomyocyte nuclei were absent from the cardiomyocyte cluster (Fig. 8J) and the proportion of cardiomyocytes in each donor was increased (Fig. 8H, K). While all cell types were identified in each sample, reflecting variability in the human population, the relative proportions of these cell types varied.

### Demultiplexing of snRNA-Seq data generated on mixed species based on alignment of reads to species-specific genomes

Genetic differences may also be used to demultiplex 10x datasets generated on samples of mixed species origin [10]. Although this method has been exploited, e.g. to determine host cell (mouse) contribution to human xenografts, it has not been used as a strategy to increase the throughput of 10x RNA-Seq analysis.

**Figure 9:**
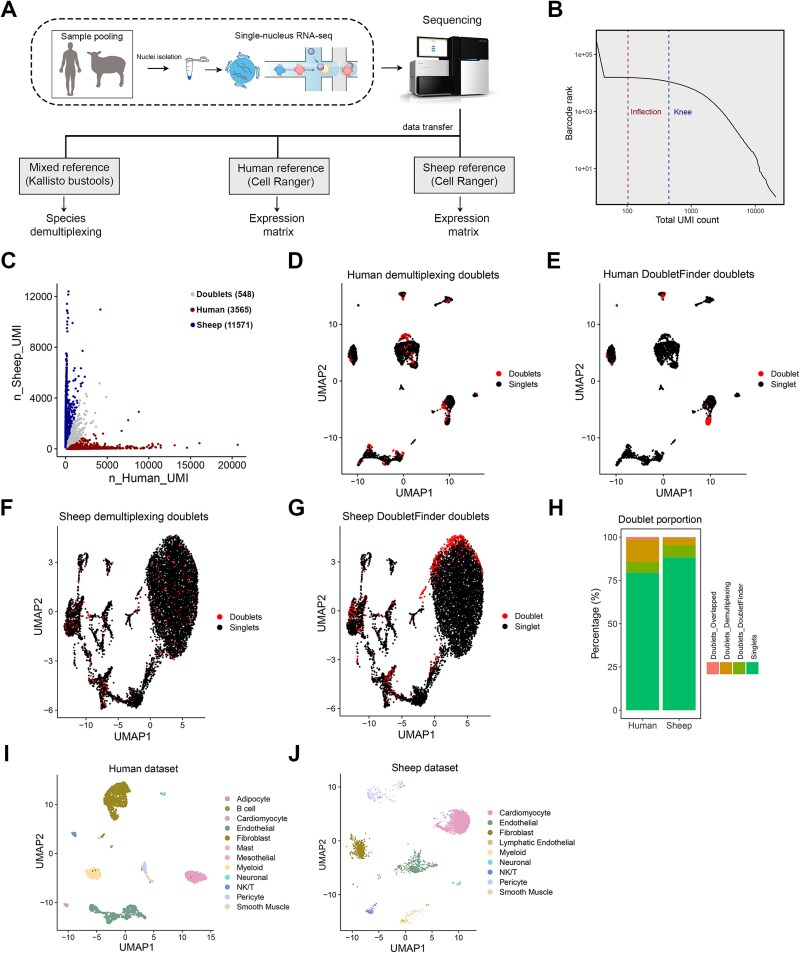
The pipeline for demultiplexing mixed-species 10x RNA-Seq dataset. (A) Schematic showing the study workflow. (B) The barcode rank plot shows the inflection point detection in EmptyDrops to assess whether the UMI associated with a cell barcode is significantly distinct from the ambient background RNA present within each nucleus. The inflection point was set as 103. (C) The scatter plot shows the number of human and sheep UMI associated with each transcriptome. Red dots and blue dots indicate the nuclei that were assigned to human and sheep species, respectively. Nuclei for which >70% of the UMIs mapped to only sheep or human were assigned to the corresponding species. (D-E) Uniform manifold approximation and projection (UMAP) representation of 423 heterogenic doublets identified by Kallisto-bustools and 223 heterotypic doublets identified by DoubletFinder in the human dataset. Doublets are highlighted in red. (F-G) UMAP representation of 423 heterogenic doublets identified by Kallisto-bustools and 687 heterotypic doublets identified by DoubletFinder in the sheep dataset. Doublets are highlighted in red. (H) The stacked bar plot depicting the proportion of each doublet type in each species. (I) UMAP representation of 12 cell types annotated by Azimuth in the human dataset (nuclei number: 2361). (J) UMAP representation of nine cell types annotated by Azimuth in the sheep dataset (nuclei number: 8085).

We therefore tested the possibility of demultiplexing human and sheep heart transcriptomes from an snRNA-Seq dataset, which was generated on a pool of nuclei isolated from 1 human and 1 sheep LV tissue sample. For this experiment, samples of equal mass of human and sheep tissue were homogenized together (Fig. 9A). The 10x sequencing reads were first mapped to a mixed reference transcriptome using the Kallisto and Bustools programs. To obtain the expression matrices for each species, the reads were also mapped separately to the human genome and the sheep genome by Cell Ranger. To obtain demultiplexing information, we first used the inflection point to eliminate empty droplets (Fig. 9B), and then based on the proportion of UMI assigned to one species, we assigned 11,571 nuclei and 3565 nuclei to sheep and human, respectively (Fig. 9C). The remainder of the 548 nuclei were assigned as heterogenic doublets. The Cell Ranger expression matrix was then used to construct the Seurat object. After removing low-quality nuclei, doublets including heterogenic and heterotypic were identified for the human (Fig. 9D, E) and sheep (Fig. 9F, G) datasets using the demultiplexing information and DoubletFinder, respectively. The proportion of doublets varied between species (Fig. 9H), with more heterogenic doublets detected in human and more heterotypic doublets in sheep. Following doublet identification, nuclei from each species were re-clustered. The human and sheep datasets were separately projected onto an annotated heart reference cell type map [39], which consists of 656,509 heart cells for cell type annotation (Fig. 9I, J). For both sheep and human, cardiomyocytes, endothelial cells, and fibroblasts were the most abundant cell types, making up 75% of the total cells between them. Despite clustering at the same resolution, the number of cell types identified was however greater in human than in sheep, where only nine were identified. Cell types absent in the sheep included adipocytes, mesothelial cells, mast cells, and B cells. These cell types are amongst the least abundant, comprising 2% of the total.

## Discussion

The emergence of snRNA-Seq technology over the past 5 years has provided a wealth of information that can be exploited to gain insights into tissue cell type composition, cell–cell variability, and cell states [40]. Yet, for large-scale comparisons, e.g. amongst patient cohorts, the cost of such an experiment remains a significant barrier leading to compromises in experimental design. Here, through benchmarking pipelines for demultiplexing of 10x snRNA-Seq data, we identify an optimal pipeline that provides high precision and recall for demultiplexing up to six samples over doublet percentage ranging from 0–30%. Further, this pipeline was computationally efficient, statistically robust, and was scalable. We further provide a full pipeline integrated with the demultiplexing tool by using a 4-sample pooled heart dataset and a pipeline for demultiplexing samples based on the proportion of UMIs for species-mixed sequencing data. These methodologies can be extended for multiplexed 10x RNA-Seq analysis of other human tissues.

While benchmarking of various tools against competitors is often reported at the time of publication, these analyses may be limited to the considered next best option, i.e., the leading competitor at the time of publication, and may be undertaken using defined criteria/package parameters and specific sample scenarios that may not reflect real-world situations. Inappropriate use of these published methods or incorrect selection of user settings can result in a bias in data analysis (such as by introducing artificial, non-biological signals) and false conclusions [41, 42]. Implementation of these software may also be a challenge in terms of computer infrastructure and expertise that preclude wide adoption. In our benchmarking analysis, these different questions were addressed. We performed a structured, comprehensive benchmark study that impartially and robustly evaluated computational methods that could be applied to demultiplex single-cell RNA sequencing data based on genomic differences. While this work is applied to the heart, the methods are fully transferable to other tissues.

Important to our benchmarking study was our access to primary 10x RNA-Seq datasets from individual and pooled samples. Moreover, the tissue from which the samples were prepared was also available, thereby allowing us to obtain information related to genetic variation, either from an SNP array of genomic DNA or expressed variants present in RNA-Seq data, which we generated using RNA prepared from tissue. In the absence of these original samples, single-cell data can be demultiplexed into anonymous individual contributors using the demultiplexing tools that we employed. However, since the identities of the samples cannot be determined, this approach dissociates other sample information from the sequencing data, thereby reducing the power of the conclusions [9]. Although limited to two samples, as shown in our analysis, sex chromosomes are also an effective strategy to demultiplex 2 samples [43], as can the mixing of species—as in a barnyard experiment [11]. The potential for using expressed variants from RNA-Seq or SNPs from arrays increases the options for investigators for demultiplexing. Bulk RNA-Seq may already be available since, e.g. they may have been generated to gain initial insights into tissue or cell transcriptome remodelling. Using these data, variant calling software can be applied to extract the expressed variants [29, 31, 32]. In the absence of this RNA-Seq data, at a similar cost, SNPs can be obtained from gDNA using SNP arrays [17]. Here, we compared the use of SNPs from these two sources for sample demultiplexing. When using expressed variants in RNA-Seq for demultiplexing, additional bioinformatical steps and computing power are required, involving variant calling tools. Here, we selected three of the most reported tools for this purpose and appended them to each of the four demultiplexing tools, creating an additional 12 separate pipelines [29, 31]. Regardless of whether SNPs were obtained from RNA-Seq or SNP arrays, Vireo performed the best in terms of accuracy (recall and precision). Souporcell also had a good performance, particularly when using SNPs from the SNP Array. Demultiplexing performance was however significantly affected by the variant caller irrespective of the demultiplexing tool used. Vireo [7] and Vireo combined with FreeBayes [32] were the best methods in terms of accuracy on the SNP array-based and bulk RNA-Seq-based datasets, respectively. The performance of Freemuxlet was particularly poor when combined with variant calling tools. The overlap of SNPs identified in the RNA-Seq data by each variant caller and those identified by SNP array varied substantially. Although the concordance of SNPs between the variant caller and array were highest with cellSNP regardless of total SNP or genic SNP, pipelines that included cellSNP data had the lowest accuracy. Among the three pipelines combined with cellSNP, two pipelines failed to demultiplex, and one achieved the lowest accuracy. Pipeline accuracy was assessed not only against a priori knowledge of sample identity and SNPs but also by testing the accuracy of the four methods to demultiplex real-world dataset comprised of 1 male and 1 female sample that could also be demultiplexed using sex chromosome-expressed genes as a ground truth. The advantage of the use of real-world datasets is the challenge offered by the presence of biological doublets and ambient RNAs, which are lacking in the simulated dataset. Importantly, the precision of nuclear identification for all methods was high, achieving an average value of 85%, which indicates all methods have the capacity to identify doublets and singlets in real-world datasets. No major differences were apparent in the performance of Vireo, Souporcell, and Freemxulet in terms of recall on the tested datasets. All obtained a good value ranging between 80% and 90%. scSplit did not however perform well on each individual. However, for one particular sample scSplit performed very poorly with a low recall value in each dataset. This is also consistent with our results on simulated datasets.

Doublets arising from the inclusion of either two independent nuclei or more nuclei that have become attached during isolation being encapsulated within a GEM resulting in the generation of a single transcriptome can, if not considered in downstream analysis, confound gene expression analysis as well as cell clustering and cell type annotation [26, 27]. Doublet removal is thus a key step in bioinformatic pipelines for snRNA-Seq data. Since doublet removal tools generally remove doublets based on the relative expression of transcripts compared to an expected distribution, in certain situations, such as for highly synthetic or polyploid cells, nuclei are more likely to be inappropriately removed. An example of this issue could arise for cardiomyocytes, which are very large cells that generate a significantly greater number of RNA-Seq reads than other cell types in the heart [44, 45]. Cell doublets may also be identified if they are genetically distinct using demultiplexing tools. Since the proportion of cell doublets comprising genetically distinct pairs of nuclei would be anticipated to rise with the number of samples combined in the sequencing pool, we tested the effects of increased doublets on the demultiplexing pipeline’s performance. Notably, the performance of each tool/pipeline decreased with increasing doublet numbers. This effect of doublet proportion was greater than increased sample number within the pool. Interestingly, and in contrast to our findings, previous benchmarking evaluations of doublet detection methods, e.g. doubletFinder, showed better performance with higher doublet percentages [46].

Important to the user is the computing run time of each pipeline. This varied substantially between pipelines ranging from 2 hours for the most rapid pipeline to 12 hours for the slowest. While it was expected that the appending of the variant caller onto each demultiplexing tool would substantially add to this run time, thus making this approach less attractive, this did not appear to be the case. Run time also scaled quite linearly with the sample number in the pool to be demultiplexed, again supporting this approach. The runtime of scSplit was, however, much longer than for the other methods, with an average time of 8.5 hours (18 cores) taken to demultiplex 6 samples. This long run time and the significant memory (~100 GB) it requires for use, especially with a higher number of nuclei (more than 10 k nuclei), would suggest that it should be avoided. The Freemuxlet method was the opposite: It had excellent robustness but medium accuracy.

Given this information, and that implementation of each pipeline is dependent on the availability of appropriate expertise and computing infrastructure as well as ease of use/documentation, we generated a scoring system that would assist in decision-making (Fig. 7). Although the overall accuracy of the demultiplexing tool is the most important aspect of evaluating the performance, computational efficiency is another important criterion to evaluate the software, which is a deficiency in previous benchmarking work [37]. Regarding overall performance, we found Vireo and Souporcell to be superior, with little difference, except for the run time, in which Vireo achieved a higher running speed than Souporcell. Freemuxlet has some good aspects, e.g. recall and robustness, but usability and precision are less optimal. The worst performer was scSplit, with no aspect of its function outperforming the other methods.

To aid researchers in integrating demultiplexing tools into their analysis, we integrated the Vireo demultiplexing tool into our standard 10x RNA-Seq pipeline and applied it to a 4-sample multiplexed dataset. Importantly, each donor in the pool was successfully demultiplexed while retaining the original donor information. This is important when analyzing the human cohort data, allowing matching with data from other sources. The human samples are varied and can be further checked after analysis. Moreover, heterogenic and heterotypic doublets identified by Vireo and DoubletFinder respectively were successfully removed. We also extended the multiplex strategy to a mixed-species experiment. By providing a pipeline applied to the human-sheep dataset, we showed that kallisto-bustools can successfully demultiplex the cells from different species based on the proportion of UMI. The removal of heterogenic doublets formed by different species also improved the performance of the analysis. In laboratories using animal models of different species, this method could provide an alternative to the use of sex or SNPs for sample multiplexing. Further, identifying the interactions between host cells (for e.g. human immune cells) and graft cells/tissues (e.g. genetically engineered organs) is important in research into cancer and xenotransplantation [47].

### Limitations

We did not include all available tools in our benchmarking analysis. We selected four widely used genetic demultiplexing tools that are representative of current mainstream approaches and which have been updated/improved since initial publication. Since the start of our analysis, additional tools such as Dropulation [48] and Demuxalot [49] have also been developed. However, due to differences in data input requirements, limited validation in the context of snRNA-Seq, or lack of widespread adoption, they were not included in our analysis. We envision that as these tools become more widely validated, our work could in the future be extended to incorporate these tools, testing them against those used in our study and assessing their performance on snRNA-Seq datasets. We also did not take the heterotypic doublets that are from different cell types into account in our simulated dataset. Although the capacity of the demultiplexing method was systematically evaluated in the simulated dataset, it also contains heterotypic doublets in practice. These heterotypic doublets can be further identified by the doublet detection method in the downstream analysis. By removing both types of doublets, the cell type annotation can be improved, which was shown in our application dataset. Therefore, future work could consider a systematic benchmarking of the doublet removal pipeline, which incorporates demultiplexing and doublet detection methods. For the demultiplexing of species, tools have been developed, e.g. for the purpose of identifying the contribution of mouse cells to human xenografts tumours [50, 51]. These tools can also be used to allow the pooling of samples, although this is dependent on sufficient SNPs in the genomes of the organism/strain analysed. With reference to the application of this method specifically to snRNA-Seq, while we demonstrate the use of this methodology to single-nucleus RNA-Seq, it is equally applicable to demultiplexing of single-cell RNA-Seq data. As we propose, the use of genetic information precludes the additional steps required for cell hashing and also provides greater accuracy.

**Key Points**

- All demultiplexing tools achieved high precision and recall, with Vireo combined with genetic information from SNP array or matched RNA-Seq exhibiting the highest accuracy.
- A pipeline that integrates the Vireo demultiplexing tool and genetic information from SNP array is provided to demultiplex real-world snRNA-Seq data from the human heart and from different species.
- The implementation of demultiplexing methods (Vireo) in a snRNA-Seq analysis pipeline leads to improvement in cell annotation and doublet identification/removal.

## Supplementary Material

Supplementary_Materials_bbaf371

Supplementary_Table_S3_bbaf371

Supplementary_Table_S4_bbaf371

## Data Availability

Bulk- and snRNA-sequencing data generated in this study have been deposited in the GEO database under accession codes GSE298265 and GSE298266. The scripts used to create the datasets in this study are available in the GitHub repository at: https://github.com/Rodericklab/Benchmarking-dmx-snRNAseq.
