# Supplementary material for: Benchmarking of computational demultiplexing methods for single-nucleus RNA sequencing data
Source: Brief Bioinform. 2025 Jul 24;26(4):bbaf371. doi: 10.1093/bib/bbaf371 (PMC12286777; doi:10.1093/bib/bbaf371)
Supplement: Supplementary_Materials_bbaf371 [file supplementary_materials_bbaf371.docx]

**Supplementary Materials**

**Benchmarking of Computational Demultiplexing Methods for Single-Nucleus RNA Sequencing Data**

Yile Fu^1^, Mohamad Youness^1^, Alessia Virzì^1^, Xinran Song^2^, Michiel R. L. Tubeeckx^3^, Gilles W. De Keulenaer^3,4^, Hein Heidbuchel^5,6^, Vincent F. M. Segers^3,6^, Karin R. Sipido^1^, Bernard Thienpont^2^, and H. Llewelyn Roderick^1,*^

^1^Experimental Cardiology, Department of Cardiovascular Sciences, KU Leuven, Leuven, Belgium

^2^Laboratory for Functional Epigenetics, Department of Human Genetics, KU Leuven, Leuven, Belgium

^3^Laboratory of Physiopharmacology, Universiteitsplein 1, Building T (2nd Floor), 2610 Antwerp, Belgium

^4^Department of Cardiology, ZNA Middelheim Hospital Antwerp, Antwerp, Belgium

^5^Research Group Cardiovascular Diseases, GENCOR, University of Antwerp, Antwerp, Belgium

^6^Department of Cardiology, University Hospital Antwerp, Antwerp, Belgium

^*^Corresponding author: H. Llewelyn Roderick, Experimental Cardiology, Department of Cardiovascular Sciences, KU Leuven, Leuven, Belgium. E-mail: llewelyn.roderick@kuleuven.be

**Supplementary Methods**

**Human samples for snRNA-sequencing data**

Left ventricular tissue samples were obtained from human hearts collected in our study of human heart failure under a protocol approved by the ethical committee of UZ Leuven (S58824), and implemented within the UZ Leuven heart transplant program. Hearts were collected and placed in ice-cold cardioplegic solution at the time of explantation. Tissue samples were taken and snap-frozen in liquid nitrogen and stored at − 80 °C.

Atrial tissue biopsies were taken from human patients during cardiac surgery, with tissue from the right atrial appendage being collected (75-250 mg per sample; Department of Cardiology, University Hospital Antwerp, Antwerp, Belgium). Informed consent was obtained from all patients. The study was approved by the institutional review committee of the University Hospital Antwerp, and the procedures followed were in accordance with institutional guidelines (EDGE001634). Samples used for sequencing analysis were snap-frozen in liquid nitrogen and stored at − 80 °C. Both study protocols conformed to the Helsinki Declaration and were conducted according to national and European Union regulations on the use of human tissues. Patient information is provided in Supplementary Table 1.

**Collection of sheep sample for sequencing**

Sheep were housed and treated according to the European Directive 2010/63/EU. Experimental protocols were approved by the local ethical committee (Ethische Commissie Dierproeven, KU Leuven), with permit number P0372022. The heart is removed under terminal anesthesia. Specifically, the sheep is first sedated using ketamine and xylazine after which general anesthesia is induced using inhaled isoflurane. Pentobarbital is then administered as analgesic followed by heparin to avoid clotting. Prior to the removal of the heart, a lethal overdose of pentobarbital is administered. The heart is then placed in ice-cold cardioplegic solution for transportation to the laboratory. Tissue samples were taken from the LV anterior walls, snap-frozen in liquid nitrogen and stored at − 80 °C.

**Nuclear isolation**

Nuclei were isolated from heart tissue by mechanical disruption, followed by centrifugation through sucrose and purification from debris by FACS as previously described [1,2]. In brief, frozen heart tissue (~100 mg) was suspended in 5 ml of lysis buffer and thawed on wet ice. When samples were pooled for 10x RNA-Seq (multiple human samples, or sheep and human), samples were either combined after (human real-world dataset) or prior to homogenization (human application dataset and sheep-human species-mixed dataset) where equivalent weights of each tissue sample were pooled. Next, samples were homogenized using a gentleMACS dissociator (Miltenyi) with gentleMACS M tubes and the Protein_01_01 protocol. 5 ml of lysis buffer containing NP-40 was added to the homogenate and incubated on wet ice for 15 min. Then, the suspension was filtered through a 30 μM Cell strainer (Miltenyi). Residual nuclei were recovered by washing the M tube and filter with 2 ml of lysis buffer containing NP-40, which was then combined with the remainder of the homogenate. The homogenate was centrifuged in a swinging-bucket centrifuge (Eppendorf, 5920R) at 1000 *g* for 5 min (4 ℃, ramp speed 4/4). The supernatant was carefully removed and the pellet containing nuclei was resuspended in 2 ml of sucrose buffer, which was then layered on top of 2 ml of sucrose buffer. The sample was then centrifuged at 500 *g* for 5 min (Eppendorf, 5920R; 4 ℃, ramp speed of 4/4) to recover nuclei. The supernatant was removed, and the pellet was resuspended in 500 μl of wash buffer and centrifuged at 500 *g* for 5 min (Eppendorf, 5920R; 4 ℃, ramp speed of 4/4). The supernatant was carefully removed and stained with 1 ml of staining buffer containing DAPI (1:1000; 7406). DAPI-positive nuclei were separated from cell debris by flow sorting using a SONY sorter instrument (SONY MA900) and collected into 50 μl of collection buffer. Sorted nuclei were then collected by centrifugation at 1000 *g* for 5 min (Eppendorf, 5920R; 4°C, ramp speed of 4/4), and the concentration was adjusted to 1000 nuclei/µl targeted for predicted capture 10,000.

**Single-nucleus RNA-sequencing library preparation**

Nuclear suspensions were loaded on a 10x Chromium Controller (10x Genomics) and processed with 10x Chromium Single Cell 3’ Reagent Kit v3 (10x Genomics) according to the manufacturer’s protocol. Cell-RT mix was prepared, targeting 10,000 cells per sample. Library quantification and quality assessment were performed using the Bioanalyzer Agilent 2100 with a High Sensitivity DNA chip (Agilent Genomics). Indexed libraries were pooled equimolarly and sequenced on an Illumina NovaSeq 6000 by GenomeScan using paired-end 26x98-bp reads as the sequencing mode. Libraries were sequenced on the NovaSeq 6000 (Illumina) at 30,000 reads per nucleus resolution.

**Bulk RNA-Seq - RNA extraction, library preparation, and sequencing**

RNA was extracted using the RNeasy Plus Universal Mini Kit (Qiagen, Germany) according to the manufacturer’s protocol. RNA quality was determined using an Agilent Bioanalyzer (Agilent, Santa Clara, CA) with samples having an RNA Integrity Number (RIN) > 7 used for library preparation. mRNAs were poly-A enriched, and after first-strand synthesis, libraries were prepared (Azenta Life Sciences, Germany). Libraries were then sequenced using Illumina’s NovaSeq 6000 with four RNA-Seq libraries per lane (2 x 150 bp paired-end reads).

**DNA isolation and genotyping**

Genomic DNA for each sample was extracted using the QIAamp DNA Micro Kit (Qiagen, Germany) and quantified by Qubit fluorometer (Thermo Fisher Scientific, USA). Extracted DNA was analyzed by Illumina Infinium Omni2.5-8 bead array (LIFE&BRAIN GmbH, Germany). SNP calling was performed using GenomeStudio software and GenTrain algorithms 2.0 with a GenCall threshold of 0.2. All samples had call rates above 98%. Samples were tested for discordance between genetically inferred sex and annotated sex, relatedness was defined via identity by state (IBS) and doublets were defined by IBS ≤ 2. The relatedness matrix is based on the PI HAT score, all calculations were done in Plink v1.9 [3].

**Benchmark evaluations workflow**

The performance of demultiplexing methods was tested on simulated multiplexed datasets, which were generated using the Snakemake [4] workflow with modifications [5]. In brief, sequencing reads (.fastq.gz) of individual samples were aligned to the GRCh38 reference genome and processed with 10x Genomics Cell Ranger 7.0.0 [6], which generated a BAM file (.bam) and gene-cell barcode count (.tsv) for each sample. Each BAM file was parsed to add unique sample IDs and merged at different sample multiplexed datasets (2 samples, 4 samples, and 6 samples) by SAMtools (.bam) [7]. At the same time, the same parse was applied to the barcode file (.tsv) and merged according to the multiplexed datasets. The lookup table (.tsv) was then generated at different doublet rates (0%, 10%, 20%, 30%) and utilized to parse and index merged BAM files to combine barcodes for doublet simulation. Finally, the candidate demultiplexing method/pipelines were applied to the simulated dataset by using either SNP array-derived or matched bulk RNA-Seq-derived genetic variation (.vcf). Table 1 is the overview summary of the demultiplexing methods in this study. The accuracy of the pipeline was evaluated in terms of Precision - the proportion of identified nuclei for each sample that are true singlet nuclei from the correct donor, and Recall - the proportion of true singlet nuclei for each sample that are identified as singlets and assigned to the correct donor.

**Benchmark environment and parameter settings**

All demultiplexing methods were executed on a server with two Intel(R) Xeon(R) Platinum 8360Y CPUs, 1024 GB of memory, and a CentOS 7.9 system. The parameters of demultiplexing methods were set to their recommended values or default values if no recommendation was available. The latest version of each method (By August 2023) was used.

**Scalability, robustness and usability**

12 snRNA-Seq datasets with varying numbers of samples (2 samples, 4 samples, and 6 samples) and different doublet rates (0%, 10%, 20%, 30%) were used to examine the scalability and robustness of demultiplexing methods. Each demultiplexing method/pipeline was applied to the 12 datasets, and the relationship between its running time and the number of samples was plotted to show the scalability, and the distributions of the mean precision and recall across subsets were plotted to show robustness, respectively.

Each demultiplexing method was evaluated as excellent, good, and fair (1, 0.5, and 0, respectively) based on 4 criteria: software quality, execution convenience, publication, and documentation & support. The ranking of a method was defined as the sum of the method’s scores in these four criteria. The software quality criterion indicates whether a demultiplexing method can be executed on all simulated and real-world datasets used in this study. ‘Execution convenience’ relates to the popularity of the computational approach required to run a method. Methods written in R and Python packages are preferred owing to their wider use in the community. ‘Publication’ relates to whether a demultiplexing method has been published in a peer-reviewed journal. ‘Documentation & Support’ evaluated a method’s user-support resources, such as open-source code, tutorials, and active Q&A.

**Species demultiplexing workflow**

Kallisto [8] and Bustools [9] programs were employed to preprocess the species-mixing snRNA sequencing data [10]. The latest release (*release 112*) of cDNA reference files from humans (*Homo sapiens*) and sheep (*Ovis aries rambouillet*) were retrieved from Ensembl [11] and combined into a hybrid indexed genome file [8]. Next, pseudoalignment of reads was performed by the Barcode-UMI-Set format (BUS) [12] using *kallisto bus* (v0.51.1) command. The BUS file generated from the previous step was further corrected using *10x v3 Whitelist Barcodes*. To reduce background noise from ambient RNA and potential UMI barcode swaps, we used CellBender remove-background with the default applied settings [13]. CellBender-corrected digital expression matrices were loaded into R as a digital gene expression matrix. The barcode ranking inflection point was used as a threshold to estimate the number of empty droplets and to consider only nuclei with the higher number of UMIs as previously described [14]. To demultiplex species, we have classified species based on the proportion of UMIs with a cutoff of 70% as previously employed [14]. Nuclei for which >70% of the UMIs mapped to only sheep or humans were assigned to the corresponding species as previously employed [14]. The remaining nuclei (those for which <70% of the UMIs mapped to only one species) were clustered as doublets.

**snRNA-Seq data QC, filtering, clustering and annotation**

Raw snRNA-Seq data were processed with the Cell Ranger pipeline of 10x Genomics to generate gene expression matrices. Species-mixed (human and sheep) data were separately mapped to both the human genome (GRCh38) and sheep genome (ARS-UI_Ramb_v2.0), whereas 4-sample-multiplexed human data were mapped to the human genome (GRCh38) only. To demultiplex the human dataset, we first used cellSNP [15] to genotype each cell, followed by Vireo [16] to assign cells to each multiplexed individual based on known genotypes. The expression matrices were then processed and analyzed using Seurat v4.3.0. Specifically, the quality control was performed in two main stages. First, nuclei assigned as heterotypic doublets (different cell types) by DoubletFinder and heterogenic doublets (different individuals/species) by Vireo or Kallisto-bustools pipeline were eliminated. Following the current best practices [17], we performed QC (<200 genes expressed and >5% of reads of mitochondrial genome origin) on each dataset separately. Nuclei outside of the threshold range of mitochondrial content, UMI counts and feature count set per subproject were filtered. Cell types were annotated by mapping the cells on a reference dataset of pre-annotated cells using the Azimuth pipeline [18].

**SNP genotype concordance analysis**

To assess genotype concordance between SNP array and bulk RNA-Seq-based variant calls, we conducted a comparative analysis across matched samples. Genotypes from SNP arrays (Illumina Infinium Omni2.5-8 bead array) were processed using the GenomeStudio pipeline and exported as VCF files. RNA-Seq-based genotypes were derived from bulk RNA-Seq BAM files using FreeBayes, BCFtools, or cellSNP, both configured to emit diploid genotype calls (GT field) for each position. We imported VCFs using the VariantAnnotation package[19] in R and extracted high-confidence biallelic SNVs. Overlapping SNPs between the array and RNA-Seq calls were identified. Genotype concordance was calculated as the proportion of SNPs with identical GT values across methods. Only positions with non-missing genotype calls in both sources were considered. Results were summarized per sample and visualized using ggplot2, stratified by SNP category (total vs. genic).

**Supplementary Figures**


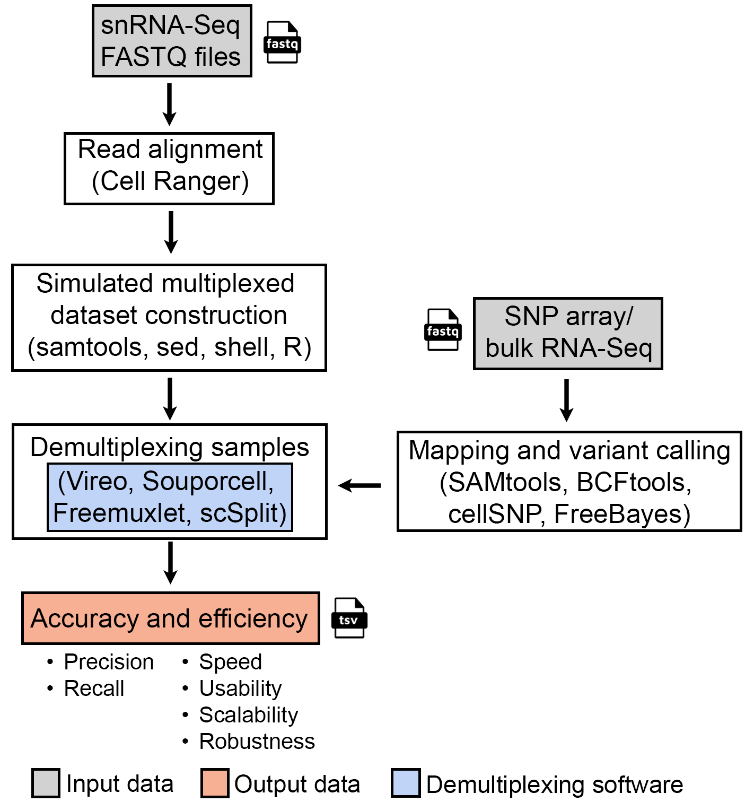


**Supplementary Figure S1. Overview of the benchmarking workflow for demultiplexing methods on simulated single-nucleus RNA-Seq data.** Raw FASTQ files from individual donor samples were aligned to the GRCh38 reference genome using Cell Ranger generating BAM files and gene-barcode count matrices. BAM files were tagged with unique sample identifiers and merged to simulate multiplexed datasets with 2, 4, or 6 donors. Corresponding gene-barcode matrices were also merged. Artificial doublets were introduced based on predefined lookup tables simulating 0%, 10%, 20%, or 30% doublet rates. These simulated datasets served as input to four demultiplexing pipelines—Vireo, Souporcell, Freemuxlet and scSplit—which utilized genotype reference data from either SNP arrays or sample-matched bulk RNA-Seq. Outputs included donor assignments and singlet/doublet classifications, which were compared against ground truth to evaluate performance using accuracy (precision and recall) and computational efficiency (speed, usability, scalability, and robustness).


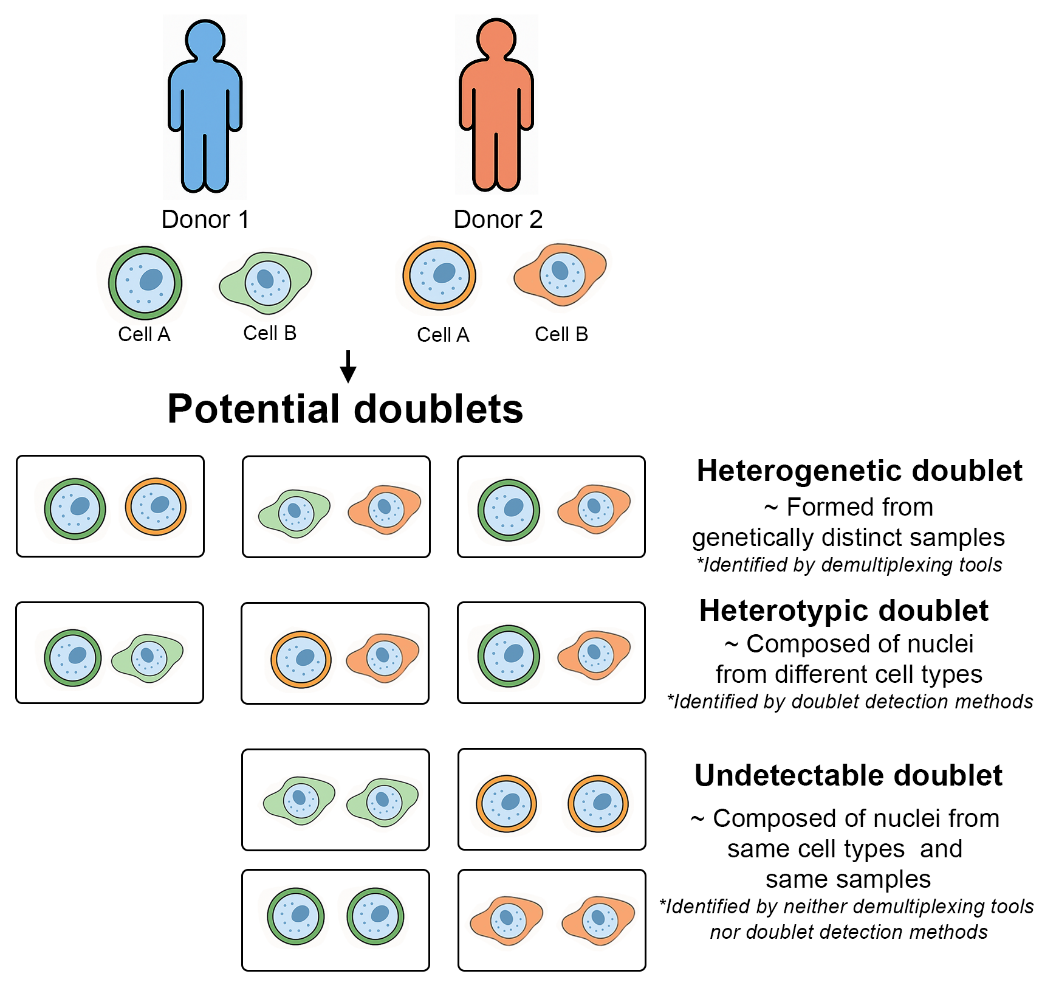


**Supplementary Figure S2. Illustration of potential doublets in the multiplexed dataset.** Heterogenetic doublets are formed from genetically distinct samples. For example, cell A from donor 1 and cell A from donor 2, cell B from donor 1 and cell B from donor 2, or cell A from donor 1 and cell B from donor 2 are potential heterogenetic doublets when donor 1 and donor 2 are multiplexed. These doublets can be identified by demultiplexing tools, such as Vireo. Heterotypic doublets are composed of nuclei from different cell types. For example, cell A from donor 1 and cell B from donor 1, cell A from donor 2 and cell B from donor 2, or cell A from donor 1 and cell B from donor 2 are potential heterotypic doublets when donor 1 and donor 2 are multiplexed. These doublets can be identified by doublet detection methods, such as DoubletFinder. Undetectable doublets are referred to as nuclei from the same cell types and the same samples. For example, two cell A/B from donor 1 or two cell A/B from donor 2 are potential undetectable doublets when donor 1 and donor 2 are multiplexed. These doublets can be identified by neither demultiplexing tools nor doublet detection methods. Notably, the number of these doublets is decreased when more samples are multiplexed.


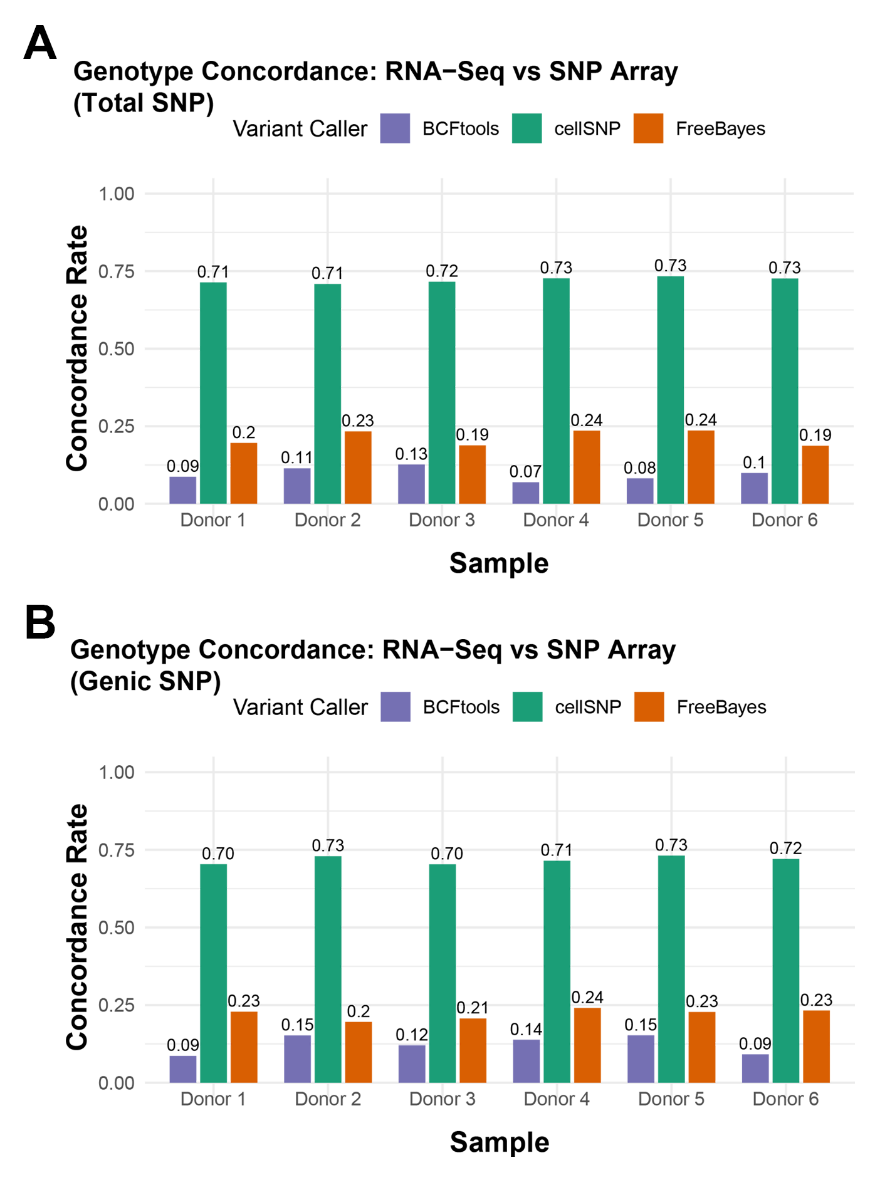


**Supplementary Figure S3. Concordance of SNP genotypes derived from RNA-Seq (BCFtools, cellSNP, and FreeBayes) and SNP array data across six donor samples based on total SNPs (A) and genic SNPs (B).** Concordance is defined as the proportion of overlapping SNPs with matching genotypes.

**Supplementary Tables**

**Supplementary Table S1.** Patient demographics and clinical information

| Sample | Diagnosis | Age  (years) | Sex | Region | Sequencing strategy  (n-sample-per-lane) | Dataset | Pooling strategy  (before or after homogenization) |
| --- | --- | --- | --- | --- | --- | --- | --- |
| Donor 1 | HF | 62 | Male | LV | 1 | Simulated | \ |
| Donor 2 | HF | 60 | Female | LV | 1 | Simulated | \ |
| Donor 3 | HF | 61 | Male | LV | 1 | Simulated | \ |
| Donor 4 | HF | 65 | Male | LV | 1 | Simulated | \ |
| Donor 5 | HF | 46 | Female | LV | 1 | Simulated | \ |
| Donor 6 | HF | 67 | Female | LV | 1 | Simulated | \ |
| Donor 7 | Non-HF | 61 | Male | LV | 2 | Real-world | After |
| Donor 8 | Non-HF | 65 | Male | LV | 2 | Real-world | After |
| Donor 9 | Non-HF | 77 | Female | LV | 2 | Real-world | After |
| Donor 10 | Non-HF | 59 | Female | LV | 2 | Real-world | After |
| Donor 11 | SR | 73 | Male | RAA | 4 | Application | Before |
| Donor 12 | SR | 73 | Female | RAA | 4 | Application | Before |
| Donor 13 | SR | 75 | Female | RAA | 4 | Application | Before |
| Donor 14 | SR | 60 | Female | RAA | 4 | Application | Before |
| Donor 15 | SR | 80 | Male | RAA | 2 | Species-mixed | Before |

HF, heart failure; SR, sinus rhythm; LV, left ventricle; RAA, right atrial appendage; Sequencing strategy, the number of samples loaded on the 10x Chromium microfluidics chip; Pooling strategy, when the tissue or nuclei were pooled together during the nuclei isolation.

Real-world dataset: one male and one female human sample; Application dataset: four human samples; Species-mixed dataset: one human and one sheep sample.

**Supplementary Table S2**. A summary table of the datasets utilized in this study

| **Dataset name** | **Description** | **Number of samples** | **Data types** | **Purpose of the study** |
| --- | --- | --- | --- | --- |
| Simulated multiplexed datasets | In silico pooled datasets with 2, 4, and 6 samples, varying doublet rates (0-30%) | 2, 4, 6  (varies per simulation) | FASTQ,  BAM | Benchmarking of demultiplexing precision and recall |
| Real-world multiplexed datasets | Single-nucleus RNA-Seq data from the human left ventricular  tissue | 2 | FASTQ,  BAM | Benchmark real-world demultiplexing accuracy |
| Application multiplexed dataset | Single-nucleus RNA-Seq data from the human left atrium  tissue | 4 | FASTQ,  BAM | Implementation of demultiplexing strategy with Vireo |
| Species-Mixed Dataset | Mixed human and sheep heart tissue for species-level demultiplexing | 2 (human + sheep) | FASTQ,  BAM | Pipeline of species-mixed demultiplexing |

**References**

1. Amoni M, Vermoortele D, Ekhteraei-Tousi S, et al. Heterogeneity of Repolarization and Cell-Cell Variability of Cardiomyocyte Remodeling Within the Myocardial Infarction Border Zone Contribute to Arrhythmia Susceptibility. Circ Arrhythm Electrophysiol 2023; 16:e011677

2. Gilsbach R, Schwaderer M, Preissl S, et al. Distinct epigenetic programs regulate cardiac myocyte development and disease in the human heart in vivo. Nat Commun 2018; 9:391

3. Purcell S, Neale B, Todd-Brown K, et al. PLINK: A Tool Set for Whole-Genome Association and Population-Based Linkage Analyses. The American Journal of Human Genetics 2007; 81:559–575

4. Köster J, Rahmann S. Snakemake—a scalable bioinformatics workflow engine. Bioinformatics 2012; 28:2520–2522

5. Weber LM, Hippen AA, Hickey PF, et al. Genetic demultiplexing of pooled single-cell RNA-sequencing samples in cancer facilitates effective experimental design. GigaScience 2021; 10:giab062

6. . Cell Ranger - Official 10x Genomics Support. 10x Genomics

7. Li H, Handsaker B, Wysoker A, et al. The Sequence Alignment/Map format and SAMtools. Bioinformatics 2009; 25:2078–2079

8. Bray NL, Pimentel H, Melsted P, et al. Near-optimal probabilistic RNA-seq quantification. Nat Biotechnol 2016; 34:525–527

9. Melsted P, Booeshaghi AS, Liu L, et al. Modular, efficient and constant-memory single-cell RNA-seq preprocessing. Nat Biotechnol 2021; 39:813–818

10. Hjörleifsson KE, Sullivan DK, Holley G, et al. Accurate quantification of single-nucleus and single-cell RNA-seq transcripts. 2022; 2022.12.02.518832

11. . Ensembl 2023 | Nucleic Acids Research | Oxford Academic.

12. Melsted P, Ntranos V, Pachter L. The barcode, UMI, set format and BUStools. Bioinformatics 2019; 35:4472–4473

13. Fleming SJ, Chaffin MD, Arduini A, et al. Unsupervised removal of systematic background noise from droplet-based single-cell experiments using CellBender. Nat Methods 2023; 20:1323–1335

14. Mereu E, Lafzi A, Moutinho C, et al. Benchmarking single-cell RNA-sequencing protocols for cell atlas projects. Nat Biotechnol 2020; 38:747–755

15. Huang X, Huang Y. Cellsnp-lite: an efficient tool for genotyping single cells. Bioinformatics 2021; 37:4569–4571

16. Huang Y, McCarthy DJ, Stegle O. Vireo: Bayesian demultiplexing of pooled single-cell RNA-seq data without genotype reference. Genome Biology 2019; 20:273

17. Luecken MD, Theis FJ. Current best practices in single-cell RNA-seq analysis: a tutorial. Molecular Systems Biology 2019; 15:

18. Hao Y, Hao S, Andersen-Nissen E, et al. Integrated analysis of multimodal single-cell data. Cell 2021; 184:3573-3587.e29

19. Obenchain V, Lawrence M, Carey V, et al. VariantAnnotation : a Bioconductor package for exploration and annotation of genetic variants. Bioinformatics 2014; 30:2076–2078
